# Modern Approach to Manage Patients With Kaposi Sarcoma

**DOI:** 10.1002/jmv.70294

**Published:** 2025-03-22

**Authors:** Thomas Bettuzzi, Celeste Lebbe, Chloé Grolleau

**Affiliations:** ^1^ AP‐HP Dermatology Department Mondor Hospital Créteil France; ^2^ AP‐HP Dermatology Department Saint‐Louis Hospital, INSERM U1342 Université Paris Cité, Diderot‐Paris VII Paris France

**Keywords:** human herpesvirus 8 (Kaposi's sarcoma‐associated herpesvirus), human immunodeficiency virus, Kaposi's sarcoma‐associated herpesvirus (human herpesvirus 8), virus classification

## Abstract

Kaposi sarcoma (KS) is a malignancy associated with Kaposi's sarcoma‐associated herpesvirus (KSHV), primarily affecting immunocompromised individuals, such as those with HIV or those receiving immunosuppressive treatments. Immunocompetent individuals may also be affected, illustrating the disease's heterogeneity. KS manifests in different forms—classic, endemic, epidemic, iatrogenic, and in men having sex with men—each with distinct clinical features depending on immune status and geographic area of origin. Although advances in treatment have improved disease control, effective management remains a challenge. This review focuses on the comprehensive approach to investigating and treating KS. It highlights the role of histology, immunohistochemistry, and staging in diagnosing KS and assessing disease extension, together with other KSHV diseases (multicentric Castelman disease, primary effusion lymphoma, and KS inflammatory cytokine syndrome). Treatment strategies are discussed, with emphasis on restoring immunity in immunocompromised patients, alongside conventional local therapies, and chemotherapy options like liposomal doxorubicin and paclitaxel for aggressive and extensive forms. Promising emerging therapies, including immunomodulatory agents, antiangiogenic therapies, and checkpoint inhibitors, are also explored. The review emphasizes the importance of personalized treatment based on the patient's underlying condition and KS subtype. It provides an in‐depth look at the pathogenesis, diagnostic methods, and evolving therapeutic approaches, offering valuable insights into improving management and outcomes for KS patients.

Kaposi sarcoma (KS) is a rare disabling disease characterized by a proliferation of Kaposi's sarcoma‐associated herpesvirus (KSHV)‐infected endothelial cells. KS was first reported by Moritz Kaposi, a dermatologist, in 1872, and the virus was identified in 1994 in KS lesions using representational difference analysis [1]. Of note, KSHV is also associated with multicentric Castelman disease (MCD), primary effusion lymphoma (PEL) [2, 3], and, more recently, with Kaposi sarcoma inflammatory cytokine syndrome (KICS) [4].

In this review, we describe the epidemiological and clinical features of KS, together with the therapeutic armamentarium with specificities according to the severity and clinical forms.

## 1 KS Forms and Epidemiology

### 1.1 Clinical Forms

There are five main clinical and epidemiological forms of KS, depending on the patient's immunologic status, and area of origin, classified as classic, endemic, epidemic, and iatrogenic KS. Epidemic and iatrogenic KS affect immunodeficient patients. Epidemic KS affects patients with HIV (PWH). Historically, it represented a progression through an AIDS stage, though it may persist despite immune restoration. In PWH, the overall risk of KS has been estimated to be more than 20 000 times greater than that of the general population and 300 times greater than that of other immunosuppressed patients [5].

Iatrogenic KS is observed in patients receiving immunosuppressive drugs, particularly after solid organ transplantation. Indeed, KS has been reported to be 200 times more frequent in transplant recipients than in the general population [6]. To a greater extent, iatrogenic KS can also be defined in immunocompromised patients affected with hematological malignancies or treated with immunosuppressive drugs for reasons other than solid organ transplantation. In these cases of iatrogenic nontransplanted KS, the most common causative drugs are corticosteroids and cyclosporine [7]. Iatrogenic KS has also been described in patients treated during long periods with topical corticoids for inflammatory skin diseases [8].

In patients without immunodeficiencies, geographic endemic areas define clinical subtypes. Although classic KS mainly affects men originated from the Mediterranean region, endemic KS affects patients from sub‐Saharan Africa. More recently, a third form of nonimmunodeficient KS has been described in non‐HIV‐infected Caucasian men who have sex with men [9, 10, 11, 12]. This clinical form has many similarities to classical KS and is now being considered as a fifth clinical variant.

### 1.2 Epidemiology

Before the AIDS epidemic, the incidence of classic KS was estimated to be higher in Mediterranean countries, with an incidence of 0.014 cases per 100 000 person‐years in the United Kingdom between 1971 and 1980 and 1.58/100 000 inhabitants per year in Sardinia between 1977 and 1991 [13, 14].

The incidence of KS then increased dramatically during the AIDS epidemic but decreased secondarily after the advent of the antiretroviral therapy (ART) era, especially in Northern countries. For example, in the United States, the incidence in PWH was estimated at 3500 cases per 100 000 person‐years in the early 1990s, compared with 500 per 100 000 person‐years in the early 2000s [15]. Similarly, a registry of 23 prospective studies in PWH reported that the adjusted incidence rate per 1000 person‐years for KS decreased from 15.2 in 1992–1996 to 4.9 in 1997–1999 [16]. Another study reported a decline in the standardized incidence ratio from 4.6 in the early 1990s to 0.3 in the late 1990s [17].

In sub‐Saharan Africa, the incidence of KS also largely increased since the AIDS epidemic. Nonetheless, in sub‐Saharan Africa, the incidence decline since the introduction of ART is less documented. Recently, a cohort using cancer registries reported a slight decline of 27% between 2000 and 2010 compared to 2011 and 2016 in KS incidence in sub‐Saharan Africa [18]. KS remains the most common cancer in men in countries such as Uganda and Zimbabwe [19]. In 2017, a large cohort of more than 200 000 patients reported that the global incidence of KS ranged from 180 to 280/100 000 person‐years in PWH from South Africa, Europe, and America [20].

The mean age at KS diagnosis varies according to the KS subtype. Although the peak incidence in classic KS is reported to be around the seventh decade of life, in endemic KS, it seems to occur 10–15 years earlier [21, 22]. In immunocompromised patients, KS onset is directly related to the contraction of HIV infection or the introduction of immunosuppressive drugs. Men are more commonly affected than women. The overall male/female sex ratio in patients with KS appears to range from 2.5/1 to 9/1, with differences across studies and clinical forms [21, 22, 23]. In classical KS, epidemic KS, and iatrogenic KS, the sex ratio is 3/1 [14, 24, 25]. In endemic KS, the male predominance seems to be higher, with a ratio of 4/1 or 5/1 [10]. Although rare, familial cases of KS have also been described [26].

Nonetheless, there is a lack of epidemiological studies assessing changes in KS epidemiology over time, between classical, endemic, iatrogenic, and epidemic KS, and in countries with limited resources.

## 2 Clinical Presentation

**Figure 1:**
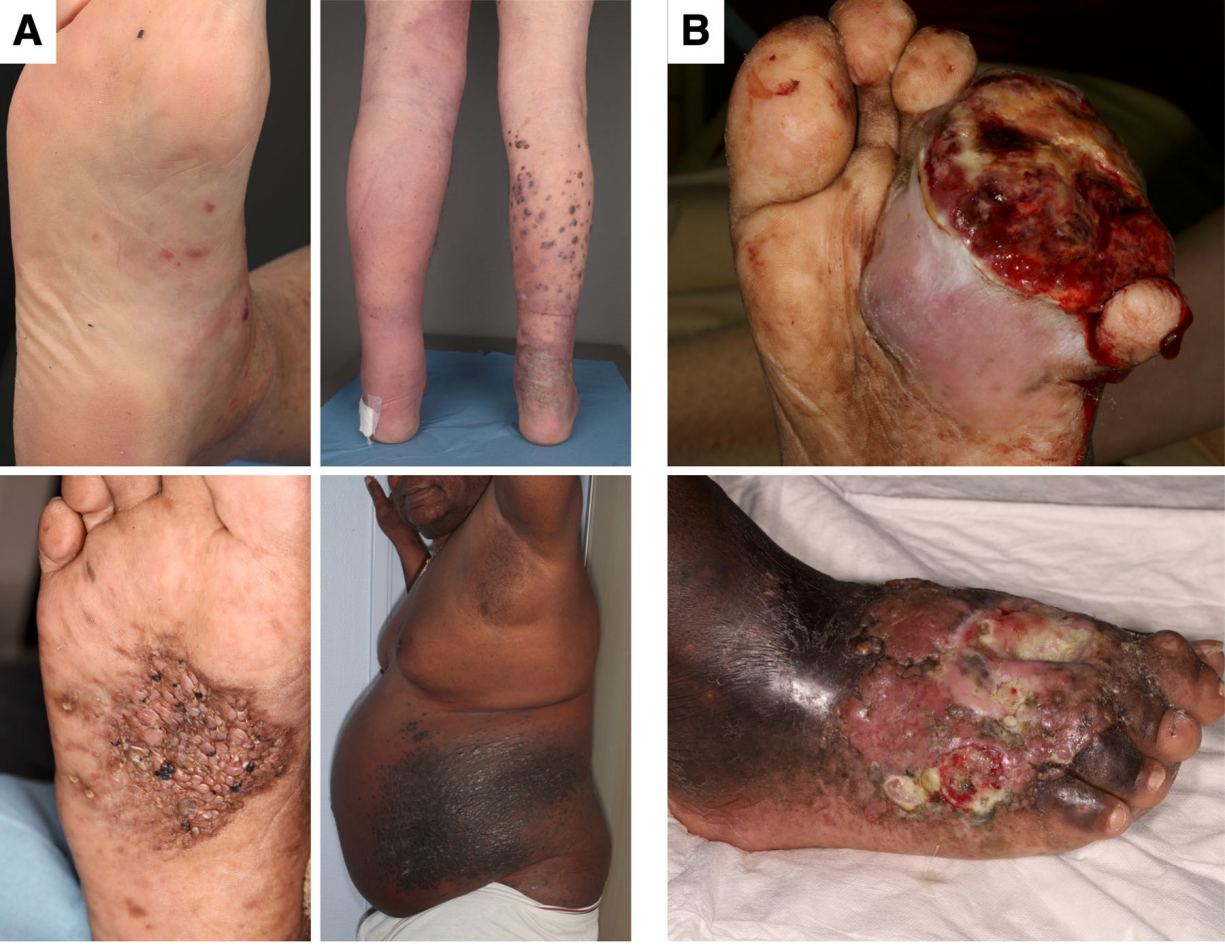
Clinical presentation of (A) localized lesions in patients with classic or endemic Kaposi sarcoma (KS) and (B) ulcerated, invasive nodules in patients with severe endemic KS.

In most cases, KS presents primarily as skin lesions. Cutaneous manifestations are characterized by purplish‐blue macules, papules, and nodules, mainly affecting the lower limbs, and are frequently associated with lymphoedema. KS may present as a single or few lesions. However, as the disease progresses, lesions may continue to appear close to or distant from the area of skin initially affected (Figure 1). Any part of the skin surface can be affected. In some cases, nodules may ulcerate spontaneously or following trauma, causing significant pain [27]. In long‐standing progressive KS, chronic edema often develops due to lymphatic dysfunction. In addition to the skin, KS can also affect mucous membranes, particularly the hard palate and, to a lesser extent, the genital mucous membrane. Of note, in our experience, ocular involvement is uncommon, but it has been reported to account for up to 20% of cases in the particular case of HIV‐related KS [28]. In cases of aggressive local extension, KS may also affect bones, most commonly in severe, ulcerative KS. Notably, rare cases of unusual locally aggressive forms of anaplastic KS have been described [29].

Apart from this local extension, systemic involvement is the main complication of KS. The most common extracutaneous KS localization is the lymph nodes [22]. More rarely, severe systemic involvement may occur, most commonly in the digestive tract and lungs [25]. Gastrointestinal symptoms differ according to the localization but may consist of dysphagia in the upper track and/or bleeding in the upper track and lower track. The severity of bleeding is also variable. Symptomatic hematemesis or melena may occur. However, gastrointestinal hemorrhage is often occult and is revealed by iron deficiency anemia. Pulmonary involvement is also a rare but well‐recognized localization, particularly in HIV‐related KS. Although frequently asymptomatic, pulmonary KS can present with dyspnea, and occasionally hemoptysis, and can be rapidly fatal if left untreated [30].

### 2.1 Clinical Presentation Within KS Subtypes

The extent of lesions and the severity of the disease are mainly related to the clinical settings of KS and the severity of immunodeficiency. In immunocompetent patients with classic KS, KS lesions are mostly localized on the lower limbs and present as macules, papules, and often as nodules that may ulcerate. Local aggressiveness can occur, but symptomatic systemic extension is uncommon. Endemic KS often occurs with the same presentation. Nonetheless, it seems to differ from classic KS with more local aggressiveness and more ulcerated nodules [22]. Of note, endemic KS had historically been described in four clinically distinct patterns, namely benign nodular cutaneous disease, aggressive localized cutaneous disease, florid mucocutaneous and visceral disease, and fulminant lymphadenopathic disease. However, this classification is almost exclusively based on reports published before the 1980s and is likely to be, at least in part, related to misdiagnosis of the HIV‐related form in patients from endemic areas before systematic HIV detection [27, 31, 32].

**Figure 2:**
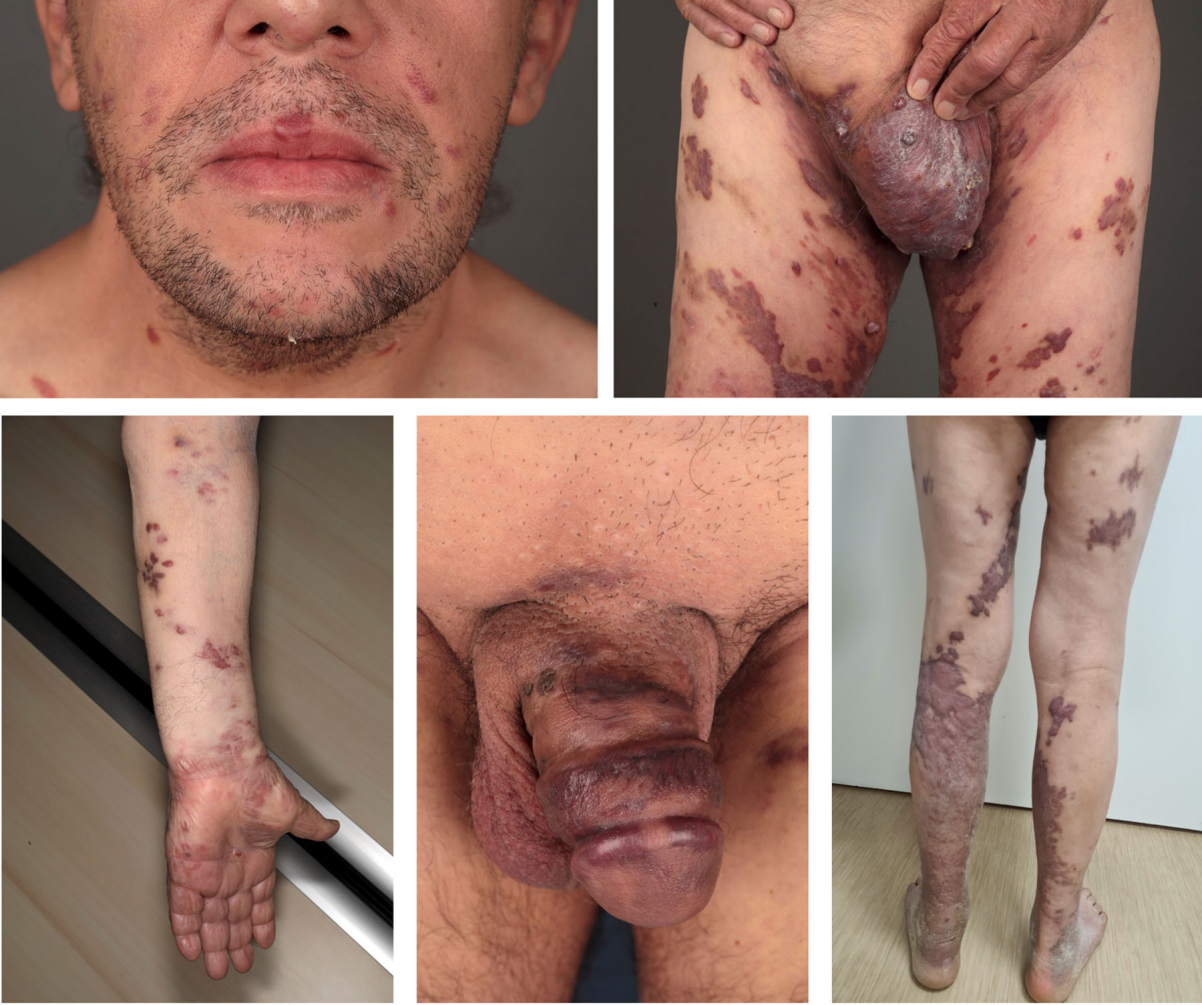
Extensive cutaneous and mucosae lesions in patients with epidemic or iatrogenic KS.

In immunodeficiency‐associated KS, the course of the disease may be chronic or rapidly progressive, depending on the alteration of the immune system. KS lesions are more likely to be extensive, associated with painful skin tumors, local skin and mucosal invasion, and systemic involvement [27, 33] (Figure 2). In epidemic KS, cutaneous lesions commonly present as multifocal and symmetric macules that may develop rapidly and evolve to papules and tumors in uncontrolled HIV infection [34]. Mucosal localizations are also frequent and may be the first manifestations of KS [28]. However, the severity of HIV‐related KS is directly related to symptomatic visceral localizations. Notably, postmortem autopsies have found evidence of visceral‐KS in up to 29% of cases [35].

Although the manifestations of HIV‐related KS are mostly related to the control of HIV infection, KS lesions may evolve independently of the course of HIV infection and persist despite controlled HIV infection and good immune system function [36]. Nonetheless, once better immunity is reached with a high CD4 count, the disease course is often much more indolent than during the AIDS stage, with a low CD4 count [37, 38].

The symptoms and lesions of iatrogenic KS are similar to those of epidemic KS and often manifest as extensive cutaneous lesions, with frequent lymph node and systemic involvement. Similar to epidemic KS, spontaneous remission after discontinuation of immunosuppressive therapy is expected, but indolent lesions may persist [24, 33].

In case of severe involvement, immunocompromised patients may develop KICS. KICS was initially described in PWH and, secondarily, in patients with solid organ transplantation [4, 39]. Conversely, it seems to be exceptional in immunocompetent patients, that is, in classical or endemic KS. As KICS was initially described as “MCD‐like syndrome” [4], the clinical features are close to MCD (see below), and KICS diagnosis supposes its exclusion.

## 3 Pathogenesis

For iatrogenic and HIV‐KS, it is known that a combination of KSHV infection and impaired immunity can cause KS. However, this is less well established for classical and endemic KS. Nonetheless, certain proteins encoded by KSHV are likely to deregulate the host immune system (see below).

### 3.1 Infection

KSHV infects endothelial cells, B cells, dendritic cells, monocytes, and fibroblasts [40]. Endothelial cells or precursors are thought to be the infected cells in KS. By contrast, MCD and PEL involve B cell infection. Similar to other herpes viruses, once the cell is infected, the virus enters a latency phase or undergoes sporadic intermittent lytic viral reactivation. However, the causes of the reactivation of KSHV remain poorly understood. This lytic cycle is thought to play a major role in the pathogenesis of MCD more than in the pathogenesis of KS. Indeed, in MCD, prevention of viral reactivation by valganciclovir or zidovudine is an effective treatment [41]. On the opposite, valganciclovir has no effect on KS [42], as, in KS, most of the cells are in the latency phase. However, a small proportion of KS‐infected cells also undergoes lytic reactivation. This fraction may have an importance, as detectable KHSV blood viremia has been associated with a worse prognosis of KS in numerous studies [43, 44, 45]. KSHV proteins and miRNA have many effects on the cellular cycle, angiogenesis, apoptosis, and immunity.

### 3.2 Effects on Cellular Cycle, Vascular Proliferation, and Apoptosis

During the latency phase, the virus expresses latent viral proteins, particularly LANA, vFLIP, kaposins as well as miRNAs. These latent genes and miRNAs have been postulated to induce tumorigenesis. Indeed, proteins encoded by KSHV affect several cellular pathways, enhancing cell survival and proliferation of virus‐infected cells, mainly the PI3K‐AkT‐mTor pathway together with the MAPK pathway and the NFkB pathway. Not exhaustively, viral proteins, including vGPCR and K1, activate the PI3K‐AkT‐mTor pathway and the MAPK pathway, leading to endothelial cell proliferation and sarcomagenesis [46, 47, 48]. vGPCR can also induce vascular proliferation in mice [46]. vFLIP, another viral protein, activates the NFkB pathway and also induces vascular abnormalities and an inflammatory phenotype in mice [49]. Moreover, K15 activates phospholipase C γ1 and induces angiogenesis [50]. K1 can also modulate AMPK function to promote cell survival [51]. In addition, the viral miRNAs inhibit apoptosis [52], and some miRNAs induce cell reprogramming.

### 3.3 Evasion of the Immune System

Many proteins encoded by KSHV are known to downregulate and evade the immune system, allowing KSHV‐infected cells to survive and proliferate. Not exhaustively, KSHV lytic proteins K3 and K5, such as MIR1 and MIR2, are known to downregulate the presentation of HLA class I [53, 54]. Viral interferon regulatory factors (vIRFs), including vIRF1, vIRF2, vIRF3, and vIRF4, are lytic proteins inhibiting the cGAS‐STING pathway and type I interferon production [55, 56]. Moreover, KSHV vIL‐6 is directly activated by IFNα and secondarily blocks interferon induction [57].

## 4 How to Investigate

The diagnosis of KS is usually evoked on compatible skin lesions. Histology is warranted for diagnosis confirmation. Of note, histology is usually performed on skin lesions. If systemic involvement is suspected, a biopsy may be performed in lymph nodes or the digestive tract. For pulmonary lesions, the benefit‐risk balance must be considered due to the subsequent risk of bleeding. Histological examination of lesional skin sample shows vascular proliferation in the dermis and extravasation of blood, resulting in the formation of hyaline globules and spindle cell proliferation, a characteristic feature of KS. Immunohistochemistry may help diagnosis, revealing the presence of spindle cells using endothelial markers like CD34, LYVE1, podoplanin, VEGFR3, or the KSHV infection using LANA staining [58]. To note, histology is similar between the different clinical forms [59].

Once the diagnosis of KS is confirmed, investigations focusing on KS extension and etiology are needed. Clinical examination should include assessment of KS severity and suspicion of extracutaneous localizations. Hence, the examination of all skin, all mucosae, including oral, especially hard palate and genital mucosa, together with lymph nodes is warranted. Asking about odynophagia, dyspnea, and hemoptysis, melena or hematemesis is also important.

**Figure 3:**
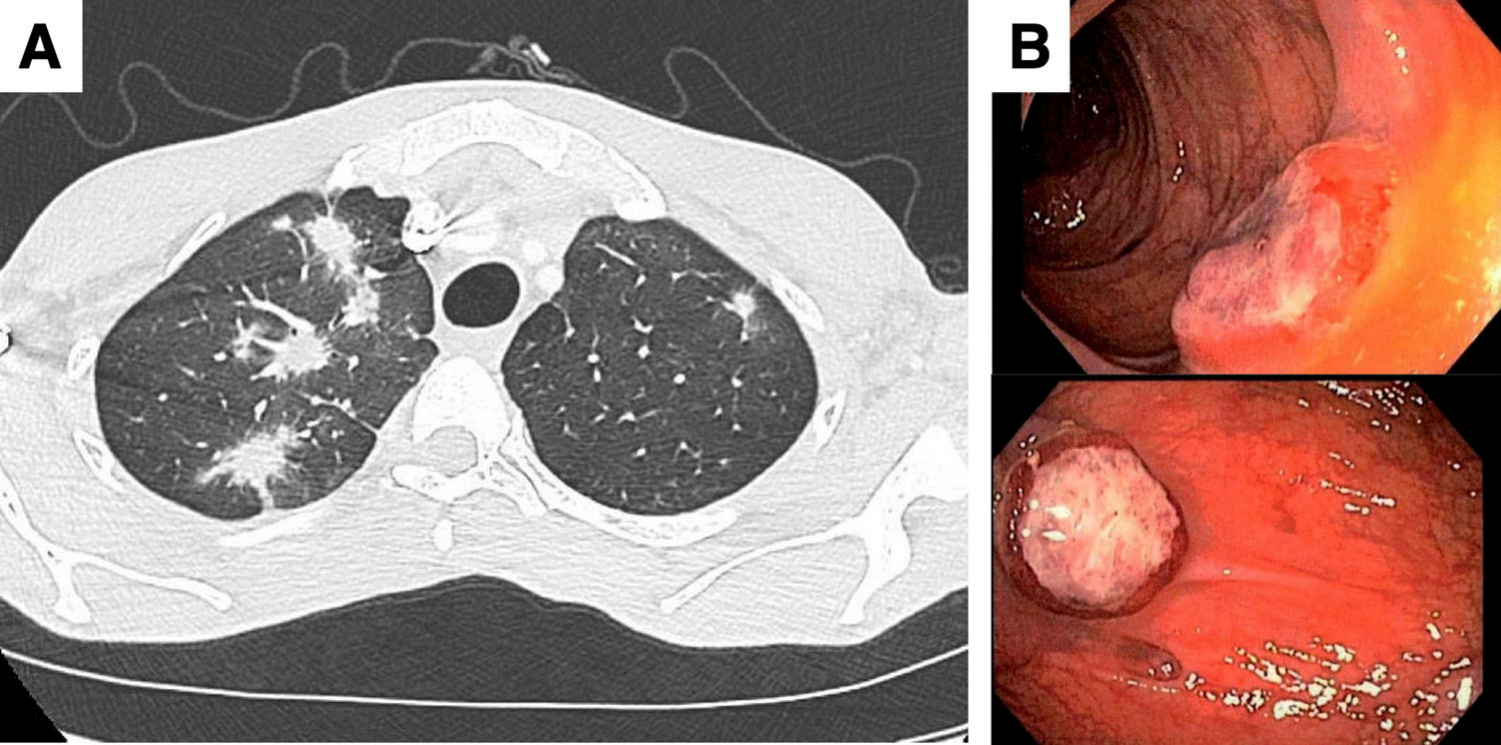
(A) Pulmonary lesion of Kaposi sarcoma identified by chest CT scan, and (B) digestive localizations of Kaposi sarcoma evaluated by colonoscopy in immunodeficient patients with invasive Kaposi sarcoma.

Secondary, additional blood tests or imaging studies may be required, depending on the clinical findings (Figure 3).

### 4.1 Complementary Exams

#### 4.1.1 KS Extension

- In all cases: screening for iron deficiency: blood count, ferritin, and C‐reactive protein.

- If deep pharyngeal involvement is suspected: ENT endoscopy.
- If digestive involvement is suspected: digestive endoscopy.
- If pulmonary involvement is suspected: chest CT scan, bronchoscopy. If biopsy is considered, the benefit‐risk balance must be considered due to the increased risk of bleeding.
- For extensive cutaneous involvement, node involvement, and suspected systemic involvement: PET‐Scan.
- If bone involvement is suspected: local scanner or local MRI, in case of need to describe the relationship with soft tissues.

#### 4.1.2 KS Etiology

- Assessment of a possible immunocompromised status: cytopenia on WBC, decreased gamma globulin levels on serum protein electrophoresis.
- HIV serology is mandatory.
- If HIV infected: CD4 count, HIV PCR.
- If transplanted: history of transplantation, immunosuppressants with dosage, and glucocorticoids with dosage.
- History of hematological malignancy.

Blood KSHV PCR is often performed. Detectable viremia has been correlated with the progression of KS in numerous publications for classic, endemic, epidemic, and iatrogenic KS, but the sensitivity and specificity of viremia in clinical practice appear to be low [43, 44, 45]. Additionally, blood KSHV PCR may be useful to detect MCD, as discussed below. Indeed, in KS, KSHV is mostly in its latent form, but in MCD, KSHV is mostly in its lytic form in B cells [41].

### 4.2 When Is Associated Castelman Disease Suspected?

Because of the common cause (i.e., KSHV infection), MCD may be associated with KS, especially in PWH. Of note, it is much rarer in other clinical forms. The symptoms of MCD are different from those of KS. MCD may be suspected, in the presence of fever, cytopenia, high gamma globulin titers, high C‐reactive protein, hypoalbuminemia, anasarca, polyadenopathy, hepatosplenomegaly, and sometimes macrophage activation syndrome. High KSHV replication in blood samples is also common [60].

### 4.3 When Is PEL Suspected?

PEL should be suspected in patients with KS who have unexplained pleural, pericardial, or peritoneal effusions, mainly in PWH [61, 62]. The diagnosis is made through serous cavity puncture or biopsy, with the identification of immunoblastic or plasmablastic cells, which typically do not express B markers (e.g., CD20, CD19) and are positively stained for LANA‐1 in immunohistochemistry [61].

### 4.4 When Is KICS Suspected?

In PWH, and to a lesser extent, in patients with solid organ transplantation [39], when there is no evidence of MCD despite extensive evaluation and, if PEL is secondarily ruled out in case of effusions, KICS should be suspected. The clinical features are close to MCD and include fever, gastrointestinal symptoms, edema, dyspnea, and anasarca [4, 63]. Similarly, biological features include cytopenia, hypoalbuminemia, high CRP, and high IL6. Of importance, KICS is also characterized by higher KSHV blood replication than usual KS.

## 5 Evolution

### 5.1 Staging

The prognosis is highly dependent on the KS subtypes, degree of immunodeficiency, and noncutaneous involvement. A staging classification for HIV associated KS was proposed in 1989 and validated in 1997, namely the AIDS Clinical Trials Group (ACTG) staging [64, 65]. HIV‐associated KS is staged according to tumor extent (T), severity of immunosuppression (I), and other systemic human immunodeficiency virus type 1 (HIV‐1) associated illness (S), and these criteria define the predicted risk of survival.

In 2003, this staging was adapted for classic KS based on four stages: macular and nodular, infiltrative, florid, and disseminated. This classification is based on the aspect and localization of skin lesions, local complications (lymphoedema, lymphorrhea, hemorrhage, pain, functional impairment, and ulceration), timing of evolution, and local/systemic aggressiveness [66]. Nonetheless, this staging has never been validated in prospective studies, and no prognosis staging is available for classic KS. As mentioned before, ACTG staging can be used in the absence of specific staging.

### 5.2 Prognosis

Importantly, although the clinical features are broadly similar, recent publications suggest a more aggressive profile for endemic KS than for classic KS. Classic KS is generally slow and indolent, and improvement without intervention or treatment is not uncommon [67]. The course of endemic KS is often more aggressive with local extensive or systemic involvement in up to 36% of cases, mainly involving bones and lymph nodes, but also visceral such as the gastrointestinal and lung localizations [68]. Recently, a cohort from our group reported that the prognosis of endemic KS was worse than that of classical KS with a lack of therapeutic response after the third line of systemic therapy. Systemic localizations and ulcerated lesions at the time of KS diagnosis were independent factors associated with a higher risk of death [22]. Furthermore, HIV‐related KS seems to be more aggressive in patients from sub‐Saharan Africa [25]. Of note, a more severe disease was reported in patients treated with immunosuppressive drugs not related to organ transplantation [7].

## 6 How to Treat

**Figure 4:**
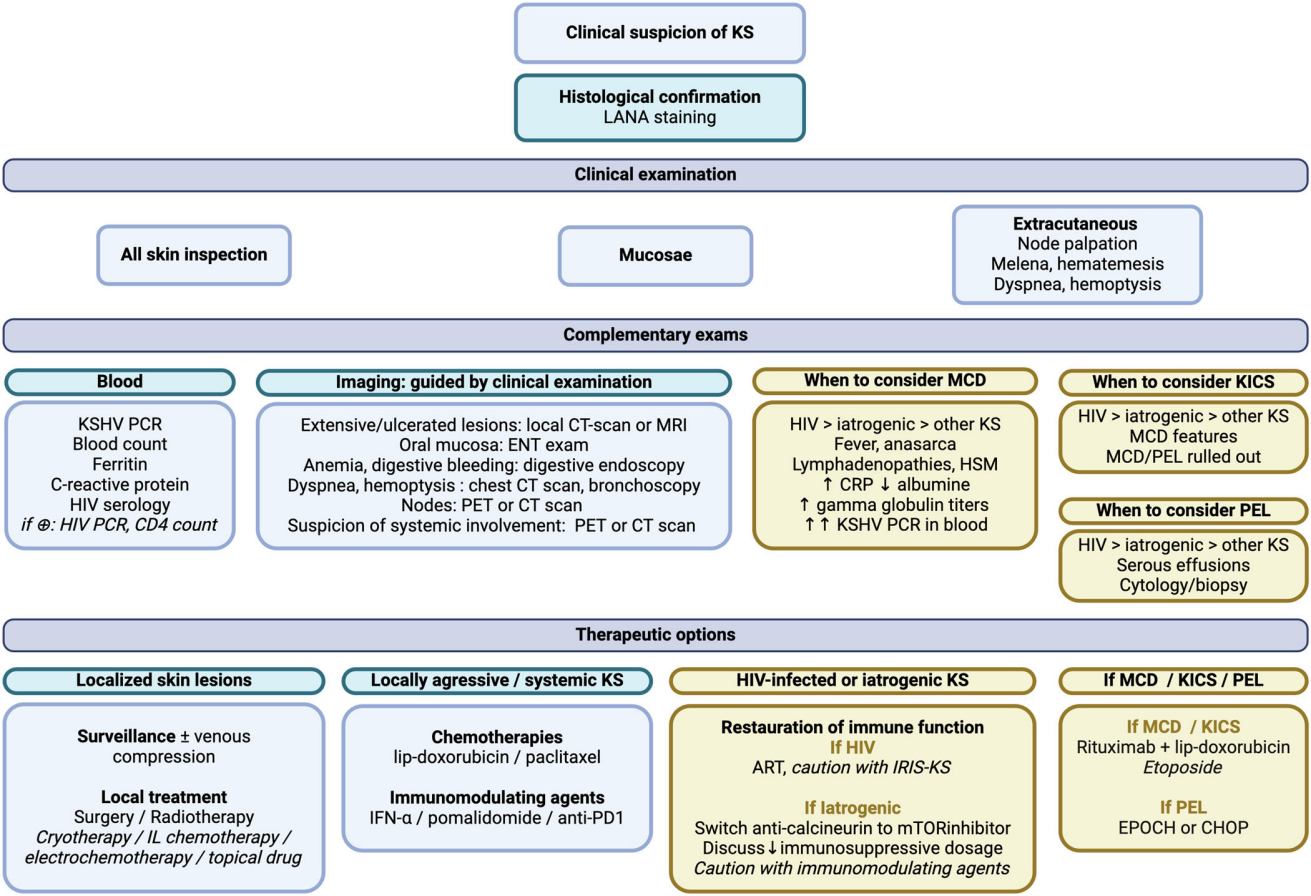
Graphical algorithm for a modern approach of Kaposi sarcoma (KS) management. ART, antiretroviral therapy; CHOP, cyclophosphamide, hydroxydaunorubicin, oncovin, prednisolone; CT, computed tomography; ENT, ear, nose and throat; EPOCH, etoposide, prednisolone, oncovin, cyclophosphamide, hydroxydaunorubicin; HSM, hepatosplenomegaly; IL, intralesional; IRIS, immune reconstitution inflammatory syndrome; KICS: Kaposi sarcoma inflammatory cytokine syndrome; MCD, multicentric Castelman disease; MRI, magnetic resonance imaging; PEL, primary effusion lymphoma; PET, positron emission tomography. Graph created in BioRender.

Restoration of immunity is the cornerstone of KS treatment, associated with a reduction in immunosuppressive drugs dosage and, when appropriate, control of HIV infection or an underlying hematologic condition. After taking into account the immunological background of the patient, specific treatment for KS may be discussed, depending on the form and the severity of KS involvement, as determined by clinical staging (Figure 4).

### 6.1 Localized Cutaneous KS

- Active surveillance and venous compression

Of importance, active surveillance is the first therapeutical option for localized KS. It may be considered in nonimmunocompromised patients, especially in classical KS due to its more indolent course and in elderly and comorbid patients [21]. Venous compression may also be helpful in case of lymphoedema [69].

- Local therapy.

If abstention is not an option, surgery may be considered in the case of isolated nodules. Alternatively, radiotherapy may be considered for large or multiple plaques or in the same area. Response rates range from 47% to 99% across studies [69]. Importantly, physicians should consider possible complications, including radiodermatitis, skin atrophy, or local infections.

Intralesional chemotherapies such as vincristine or vinblastine are also historical approaches that have shown partial to complete responses in limited KS, with reported efficacy ranging from 70% to 98% of cases [70, 71]. Electrochemotherapy, a strategy combining intralesional chemotherapy and electroporation, is also an interesting option for localized cutaneous KS [72].

For limited lesions consisting mainly of macules and papules, cryotherapy or local imiquimod 5% may also be considered, with a response rate of 40% for imiquimod [73, 74].

### 6.2 Severe Cutaneous Lesions or Extracutaneous Manifestations of KS

#### 6.2.1 Chemotherapy and Conventional Therapy

Liposomal doxorubicin is considered to be the first‐line chemotherapy for advanced KS. In the 1990s, several randomized controlled trials (RCTs) conducted in HIV‐related KS demonstrated its superiority over conventional cytotoxic therapies such as bleomycin and vincristine [75, 76, 77]. In epidemic KS, RCTs have shown the response rates ranging from 46% to 58%.

Paclitaxel is the other chemotherapy that demonstrated its efficacy in RCT conducted in PWH, with overall response rates ranging from 56% to 71%, either in first‐line or second‐line treatment after liposomal doxorubicin [78, 79, 80]. In a recent RCT in 2020, paclitaxel was more effective than etoposide monotherapy and bleomycin and vincristine combination in PWH [81].

Nonetheless, RCTs comparing systemic treatments in patients with KS are lacking. Moreover, most of the trials evaluating the efficacy of systemic therapies were conducted before the modern ART era. In PWH, only one head‐to‐head RCT was conducted for liposomal doxorubicin and paclitaxel and reported similar efficacy, with a better tolerability for liposomal doxorubicin [82]. Therefore, due to this better tolerance, the first choice is often liposomal doxorubicin in resource‐rich settings. Conversely, in resource‐limited countries, the first choice is often paclitaxel, in line with its better efficacy than other conventional chemotherapies.

Thereafter, within variable delays after treatment cessation and despite initial response, recurrence and, subsequently, retreatment is common. In case of initial effectiveness, the same treatment regimen can be used. Nevertheless, cumulative toxicity should be closely monitored, that is, cardiac function for liposomal doxorubicin and neurotoxicity for paclitaxel.

Other chemotherapies may be considered as second‐line alternative therapies in patients who are refractory or have contraindications to liposomal doxorubicin or paclitaxel. Other alternative chemotherapies include vinblastine, bleomycin, gemcitabine, and etoposide [83, 84, 85, 86, 87].

To note, low‐dose recombinant interferon alpha (IFNα), an old immunomodulatory treatment, might be considered in locally extensive forms of classic and endemic KS without extracutaneous manifestation [88, 89]. Only one small sample RCT directly compared liposomal doxorubicin and low‐dose IFNα in classic KS, with observed major response of 92% and 84%, respectively [90]. A recent, retrospective, but larger study in classic and endemic KS patients showed lower responses, with an overall response observed in 56% of KS and a probable 51% rate of adverse events.

Of importance, the evaluation of conventional therapies in non‐HIV‐related KS patients is based on retrospective cohorts and centers’ experience and is derived from RCTs conducted in PWH. To note, recently, a retrospective multicentric study reported the efficacy of liposomal doxorubicin and paclitaxel in classic and endemic KS with similar response rates of approximately 80% for both arms [22].

#### 6.2.2 Immunomodulatory and Antiangiogenic Agents

Pomalidomide received Food and Drug Administration approval in 2020 for the treatment of patients affected with KS without HIV infection or for HIV‐related KS after failure of antiretroviral therapy. A clinical trial conducted in 28 patients with and without HIV infection reported an overall response rate of 71%. The most frequent adverse event was neutropenia, particularly Grade 3 in up to 50% of patients [91, 92]. Of note, a Phase I study explored the effect of pomalidomide associated with liposomal doxorubicin in KS patients, with a partial response rate of 81% [93].

Other antiangiogenic and immunomodulatory agents such as lenalidomide have shown in vitro efficacy in restoring the immune phenotype on KSHV‐infected cells [94]. However, lenalidomide was a failure in PWH with KS with no complete response nor partial responses in a nonrandomized Phase II trial in 12 patients in France [95]. Conversely, a recent Phase I/II trial in 15 and 23 PWH reported a response in 60% of patients in the United States [96].

##### 6.2.2.1 Severe cutaneous lesions or extracutaneous manifestations of KS

In recent years, checkpoint inhibitors, particularly anti‐PD1, have shown promising activity in KS patients. A clinical trial conducted in 30 nonimmunodeficient KS patients treated with pembrolizumab showed an overall response in 71% of cases. In HIV‐related KS, two studies focusing on HIV‐related cancer suggested a good efficacy of anti‐PD1 with an acceptable safety profile [97, 98]. More recently, a Phase I trial in patients with epidemic KS reported a 62.1 response rate in patients with KS [99].

### 6.3 Specific Management of KS Subtypes

#### 6.3.1 Specificity of Management in Patients With Classic and Endemic KS

Whenever possible, active surveillance or local treatment should be preferred, given the generally benign course and even the potentially self‐resolving nature of skin lesions in these KS forms (mostly classic KS). However, in the case of locally advanced, hyperalgesic lesions or extracutaneous manifestations, a systemic treatment may be warranted. As mentioned above, most RCTs have been conducted and designed for HIV‐related KS, and their conclusions have been derived for classic and endemic KS. Recently, in a multicenter retrospective study, liposomal doxorubicin and paclitaxel appear to be equally effective in classic and endemic KS, with a better tolerability profile for liposomal doxorubicin [22]. IFNα, although effective in some patients, tends to be associated with lower response rates than paclitaxel and liposomal doxorubicin [22].

#### 6.3.2 Specificities of Treatment in Transplant Recipient Patients

Collaboration between the “KS” physician and the “transplantation” physician is essential. If possible, the dose of immunosuppressants is reduced, and/or calcineurin inhibitors are changed to mTor inhibitors. Indeed, there is a rationale to use sirolimus because the mTor pathway is activated by KSHV [100]. In the pivotal trial of 15 kidney transplant recipients with KS, all KS lesions disappeared after switching from a calcineurin inhibitor to sirolimus [101]. More recently, a retrospective cohort study in 145 transplant recipients with KS reported that, although conversion to mTor inhibitors is more likely in patients with systemic involvement, reduction of immunosuppression along with a switch to mTor inhibitors allows KS to stabilize or respond in most patients [24].

Importantly, mTor inhibitors should not be the systemic treatment for other clinical forms, mainly classical and endemic KS. Indeed, in a Phase II clinical trial of 11 patients with endemic or classic KS who received everolimus, dissociated responses were noted [102]. Target KS lesions were reduced, but the appearance of new lesions was noted in many patients. A possible explanation for this dissociated response was that everolimus inhibited the mTor pathway and reduced KS lesions, but its immunosuppressive profile led to the development of new KS lesions.

Because of the subsequent risk of allograft rejection, immunomodulators must be used very cautiously, especially IFNα or immune checkpoint inhibitors. A report based on an analysis of the pharmacovigilance database highlighted a high risk of rejection in organ transplant recipients treated with checkpoint inhibitors, which appeared to be increased with combination anti‐PD1 and anti‐CTLA‐4 therapy [103]. Hence, due to the possibility of extrarenal epuration, anti‐PD1 could be considered for kidney transplant recipients with severe and refractory KS but remains very risky for other organ recipients, mainly liver, heart, or lung, with organ rejection likely to cause death. In kidney transplant recipients with severe and/or refractory KS, detransplantation with progressive reduction of the immunosuppressive treatment dosage and return to extrarenal epuration is an option. Logically, this therapeutic option is not appropriate in patients with liver, heart, or lung transplants.

#### 6.3.3 Specificities of Management in PWH

Restoration of the T cell immune system with ART is the first‐line therapy for the treatment of KS. However, improvement may be slow and may take several months. In cases of life‐threatening localization (e.g., gastrointestinal bleeding and pulmonary lesions with hemoptysis) that cannot wait for immune system reconstitution, systemic treatment may be administered. The first choice is pegylated liposomal doxorubicin because of its limited immunosuppressive profile compared with paclitaxel and overall better tolerability [82, 104]. In addition, glucocorticoids are in general administered with paclitaxel at least in the initial courses and may theoretically interfere with immune reconstitution.

Of note, a proportion of PWH‐related KS may experience a worsening of their clinical lesions after ART onset, known as the KS immune reconstitution inflammatory syndrome (KS‐IRIS). Despite an initial decline of CD4 count in these patients, it was reported that ART should be continued [105]. Nonetheless, KS‐IRIS is a reason to begin systemic therapy in PWH with low CD4 count and aggressive disease or extensive KS.

#### 6.3.4 How is KS Associated With Castelman Disease Treated?

As stated below, KS may be associated with MCD, particularly in PWH. Importantly, rituximab, a major component of MCD treatment, has been associated with flare‐ups of KS [106], so great care must be taken in case of underlying both MCD and KS if rituximab is administered. If MCD and KS coexist, rituximab and liposomal doxorubicin should be given together [107]. Etoposide is frequently given as an adjunct because it is effective in both diseases and, in particular, allows rapid control of KHSV lymphoproliferation and macrophage activation syndrome [81, 108, 109]. However, it should be noted that etoposide is less effective than liposomal doxorubicin in patients with KS and that no trials have been conducted yet to evaluate the adjunction of etoposide to liposomal doxorubicin in patients with both KS and MCD.

#### 6.3.5 How is PEL Treated

PEL is a rare KHSV‐associated lymphoma that needs to be managed by care in specialized centers. Treatment typically involves a combination of chemotherapy, including EPOCH (Etoposide, Prednisolone, Oncovin, Cyclophosphamide, Hydroxydaunorubicin) or CHOP (cyclophosphamide, hydroxydaunorubicin, oncovin, prednisolone), often in combination with rituximab to treat concomitant MCD [1, 2, 21, 61, 110]. The place of daratumumab in the therapeutic strategy of PEL is yet to be further investigated [111, 112].

#### 6.3.6 How is KICS Treated?

Data regarding KICS treatment are very limited. However, KICS management resembles that of MCD more than KS treatment. With this in mind, it is important to note that KS should be treated first, mainly with liposomal doxorubicin, to avoid any risk of KS flare due to immunosuppressive drugs. Additionally, adjunctive treatments for KICS may be introduced. Rituximab and, to a lesser extent, etoposide or valganciclovir and zidovudine might be considered [113, 114]. To our knowledge, although interleukin‐6 seems to be a key cytokine in KICS pathogenesis, there have been no reports on the efficacy of tocilizumab.

## 7 Perspectives

KS is a complication of KSHV infection that affects only a proportion of those infected, and the underlying mechanisms remain largely unknown. Although KSHV infection together with immunocompromised status is known to contribute to the onset of epidemic and iatrogenic KS, the triggers for classic and endemic KS remain poorly understood.

Multiple infections with different strands of KSHV, together with pathogenic viral protein polymorphisms of interest, acting as oncogenes, appear to be a possible explanation for the development of KS following KSHV infection [115]. However, host factors remain unknown and still have to be unraveled. A better understanding of KS pathogenesis is essential to improve therapeutic options.

Over the past decade, treatment options for KS patients have improved, enabling satisfactory disease control in many cases. However, these advanced therapies are mostly available in Western countries. The burden of KS falls disproportionately on African countries where diagnosis and treatment options are limited. In these regions, access to HIV diagnosis and treatment remains challenging. Access to newer treatments such as pembrolizumab or pomalidomide, as well as older but effective therapies such as liposomal doxorubicin, is critical for these countries.

In addition, as herpes papillomavirus (HPV) for cervix cancer, the development of an effective vaccine [116] against KSHV could dramatically reduce the incidence of KS in endemic regions and should be encouraged [117].

## Ethics Statement

Informed written consent was obtained from all patients for the publication of their images.

## Conflicts of Interest

The authors declare no conflicts of interest.

## Data Availability

The authors have nothing to report.
